# Development and evaluation of mouse anti-Ara h 1 and Ara h 3 IgE monoclonal antibodies for advancing peanut allergy research

**DOI:** 10.1016/j.mex.2023.102470

**Published:** 2023-11-04

**Authors:** Takaki Waritani, Sidney Lomax, Dawn Cutler, Jessica Chang

**Affiliations:** Chondrex, Inc., 16928 Woodinville-Redmond Rd NE STE B101, Woodinville, WA 98072, USA

**Keywords:** Peanut allergens, Allergy, IgE, Mast cells, Hypersensitivity, ELISA, Immune-blot, Development and Evaluation of IgE Monoclonan Antibodies.

## Abstract

Immediate hypersensitivity reactions to peanuts are a considerable public health concern due to the acute and severe IgE mediated reactions. To conduct research on the pathogenesis and therapeutics of peanut allergies, it is imperative to have mouse anti-crude peanut extract (CPE) IgE monoclonal antibodies (mAbs) for both *in-vitro* and *in-vivo* assays. Without these tools, it is difficult to advance research in this field.

In this study, four hybridomas producing anti-CPE IgE mAbs were developed and the IgE mAbs were validated using immune-blot analysis, Sandwich ELISA, Indirect ELISA, a cell-based assay using RBL-2H3 cells, and footpad type I hypersensitivity reaction studies in mice.

The results indicate that two of the four mAbs can be effectively used for both *in-vitro* and *in-vivo* peanut allergy studies, as they induce allergic reactions with sensitization alone in mice. These novel anti-Ara h1 and Ara h 3 IgE mAbs, in combination with the detailed protocols outlined in this article, offer valuable guidance for studying acute allergic reactions involving mast cells across various platforms. With some considerations, the IgE mAbs can significantly advance peanut allergy research.

## Graphical abstract

**Figure:**
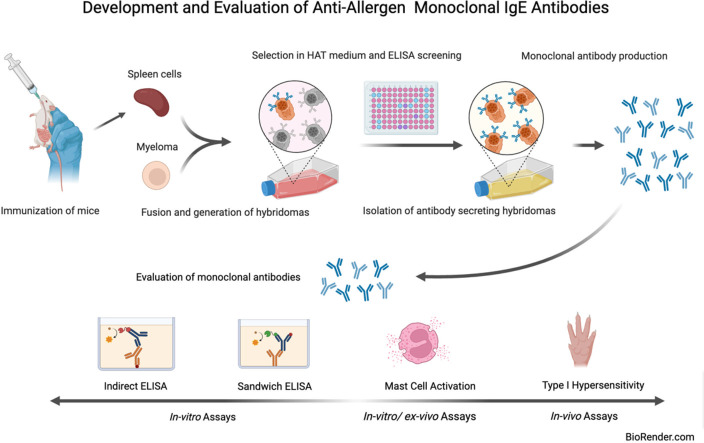

Specifications table

**Table:** 

Subject area:	Immunology and Microbiology
More specific subject area:	Peanut allergy research
Name of your protocol:	IgE mAb related *in-vitro* and *in-vivo* peanut allergy studies
Reagents/tools:	See Methods section
Experimental design:	IgE monoclonal antibodies against peanut allergen, Ara h 1 and Ara h 3 were validated by immuno-blot, sandwich ELISAs, indirect ELISAs, cell-based assays, and type I hypersensitivity studies in mice.
Trial registration:	None
Ethics:	All animal experimental protocols were approved by the Animal Experiment Committee of University of Washington.
	This study used female BALB/c mice.
Value of the Protocol:	Development of IgE antibodies
	*In-vitro* assays for validating IgE antibodies
	*In-vivo* assays for validating IgE antibodies

**Description of protocol**

## Introduction

The peanut allergy, an IgE-mediated food allergy, is a major public health concern, particularly in westernized countries. Food allergies affect 10 % of the US population, with over 6 million people, or 2 % of the population, experiencing peanut allergies [1,2]. These immediate hypersensitivity reactions to peanuts persist into adulthood. For peanut-allergy patients, avoidance currently remains the viable option [3], but for children ages 4-17, oral immunotherapy (Palforzia) has been approved by FDA in 2020 [4]. Individuals affected with peanut allergies are at greater risk of developing anaphylaxis compared to those with other allergies [5,6].

Seventeen potentially peanut allergens have been identified [7]. Of these allergens, Ara h 1, Ara h 2, Ara h 3, and Ara h 6 have been designated the major peanut allergens. Ara h 2 and Ara h 6, two highly related 2S albumins, especially contribute to the development of allergic reactions [8].

Mouse peanut allergy models have been used to study the pathogenesis of the peanut allergy and to develop new therapeutics. The models can be induced by the administration of crude peanut extract (CPE) or each purified Ara allergen and evaluated for the humoral immune responses such as serum anti-IgE and IgG antibodies against the allergens, cytokines levels associated T-cell mediated immune responses, as well as changes in body temperature, and clinical signs of anaphylaxis. These changes observed in the disease models are useful for studying the efficacy of treatments in preventing allergic reactions [9], [10], [11], [12], [13], [14], [15].

There are several protocols for developing mouse peanut allergy models, such as a combination of an aluminum hydroxide adjuvant and oral administration [16,17], oral administration only [8,[12], [13], [14], or intranasal administration [9]. However, developing these models can take 4-8 weeks as the immune system needs time to induce IgE antibodies against peanut allergens. Additionally, serum IgE levels against allergens in mice can vary greatly depending on the doses of the allergen, sensitization routes, mouse strains, and protocols used [18], [19], [20].

In the development of peanut allergies, anti-allergen IgE antibodies play a crucial role in inducing allergic and anaphylaxis reactions by activating mast cells (MCs) [21]. IgE antibodies can bind to two types of IgE receptors: the high-affinity FcεRI and the low-affinity FcεRII. FcεRI is primarily expressed on MCs, basophils, and dendritic cells. Crosslinking of FcεRI by immunocomplexes of IgE antibodies and allergens can activate the receptors and initiate intracellular signaling pathways. The activation results in both cell degranulation and the release of preformed mediators such as amines, proteoglycans, proteases, lysosomal enzymes, newly formed lipid mediators, cytokines, and chemokines (GM-CSF, IL-1b, IL-8, IL-13, MCP-1) [22,23]. In fact, inhibitors of MCs activation have been clinically tested in an attempt to reduce allergic responses [22,24].

Although IgE monoclonal antibodies (mAbs) against various antigens have been developed for MCs assays [25,26] and animal studies [27], no mouse IgE mAbs against peanut allergens have been reported although human IgE mAbs have been established [25]. Furthermore, the levels of IgE antibodies against peanut allergens can vary greatly even among animals in the same cage and affect the incidence and severity of allergic reactions. To advance peanut allergy research, we have developed mouse anti-CPE IgE mAbs and evaluated their specificities and biological activities in *in-vitro* and *in-vivo* studies. These novel anti-CPE IgE mAbs, along with the protocols outlined in this article, offer valuable guidance for studying allergic reactions across various platforms, thereby advancing peanut allergy research.

## Methods

### Crude peanut extraction preparation

Crude peanut extracts (CPE) were prepared by modifications of a published protocol [28]. Raw peanuts were manually ground using a coffee grinder. The peanut powder was then suspended in a TBS buffer (65 mM Tris-HCl, 1 mM EDTA, 1 mM PMSF, 200 mM NaCl, pH 8.3) at a ratio of 2:100 (w/v) and stirred for 1 hour at room temperature (RT). The extract was filtered through Grade GF/A filters (Whatman plc, United Kingdom), and then centrifuged at 10,000 × g for 30 minutes at 4°C. The supernatant was dialyzed against distilled water and lyophilized. The lyophilized powder was dissolved in TBS buffer. This solution was taken to 40 % ammonium sulfate saturation and centrifuged at 30,000 × g for 30 minutes at 4°C. The resulting supernatant was then taken to 70 % ammonium sulfate saturation and centrifuged at 30,000 × g for 30 minutes at 4°C. The pellet was resuspended in TBS buffer [29]. Batches of crude peanut extract (CPE) (31–33 mg/ml) were clarified by centrifugation and stored at −70°C. Protein concentrations were determined using the Pierce BCA kit (Pierce, Rockford, IL, USA) using BSA as the standard [28].

### Intragastric sensitization and challenge

Six-week-old female BALB/c mice were purchased from Envigo (Bar Harbor, ME) and housed under specific pathogen-free (SPF) conditions. To obtain sufficient mice for somatic cell hybridization, a group of five mice were housed together in a cage, and each mouse was sensitized by intragastric gavage with 5 mg of CPE and 10 µg of cholera toxin (List Biological Laboratories, Inc, CA, USA) in 0.5 ml of Phosphate Buffered Saline, pH 7.4 (PBS) per mouse on day 0 and again on day 7. Three weeks after the initial sensitization, the mice were fasted overnight and challenged with 10 mg CPE in 0.5 ml PBS, given in two doses separately with 30-40 minutes in between, through intragastric gavage [11]. Serum samples were collected and stored at –20°C. The animal experiments were conducted in accordance with the guidelines for animal use and experimentation set out by our institutions.

### Development of IgE monoclonal antibodies against CPE

Monoclonal antibodies (mAbs) were produced through the technique of somatic cell hybridization. Briefly, BALB/c mice received a booster injection intravenously with 10 µsg CPE dissolved in 0.1 ml PBS, after intragastric sensitization and CPE challenge. Three days after the booster injection, the mice were sacrificed, and their spleens were removed to prepare single cell suspensions in DMEM medium. These spleen cells were then fused with SP2/0-Ag4 cells at a ratio of 5:1 using 50 % polyethylene glycol (MW.4000). The fused cells were plated onto 96-well flat-bottomed tissue culture plates (2.0×10^5^ cells/well) and cultured in DMEM medium supplemented with 15 % fetal calf serum (FCS) containing hypoxanthine, aminopterin and thymidine in a humidified atmosphere of 7 % CO_2_ in air at 37°C. All plates were screened for anti-CPE IgE antibody-producing hybridomas by ELISA kits (Cat# 3063, Chondrex, Inc. WA, USA), according to the manufacturer's instructions. Hybridomas producing anti-CPE IgE mAbs were cloned through the limiting dilution method [30]. The mAbs produced by hybridomas were purified through DEAE ion-exchange chromatography (GE Healthcare Systems, Chicago, IL,USA) from culture medium collected with the miniPERM culture system (Sarstedt, Newton, SC, USA) [31].

### Anti-CPE IgE antibody sandwich ELISA

Anti-CPE IgE mAbs were assayed using ELISA kits (Cat# 3063, Chondrex) following the manufacturer's instructions.

### Anti-CPE IgE antibody Indirect ELISA

96-well high-binding ELISA plates (Greiner Bio-One, NC, USA) were coated with 1 µg/well in PBS at 4°C overnight. After washing the plates with PBS containing 0.05 % Tween 20 (PBST), the plates were blocked with PBS containing 1 % bovine serum albumin (BSA: Roche Diagnostics, IN, USA) (BSA/PBS) for 1 hour at RT. After washing the plates with PBST, anti-CPE IgE mAbs diluted with BSA/PBS were added to the plates and incubated for 2 hours at RT. After washing the plates with PBST, biotin-labeled anti-mouse IgE monoclonal antibodies (Chondrex) diluted in 0.05M Tris-buffered saline buffer, pH 8.0 containing 2 % Casein (Sigma-Aldrich, Inc., MO, USA) and 0.05 % Tween 20 (Casein/TBS) were added to the plates and incubated for 1 hour at RT. After washing the plates with PBST, HRP-labeled streptavidin (Chondrex) in Casein/TBS was added to the plates and incubated at RT for 30 minutes. After washing the plates with PBST, the color was developed at RT for 25 minutes by adding 100 µl of 3,3′,5,5′-tetramethylbenzidine solution (TMB, Chondrex). The enzymatic reaction was stopped by adding 50 µl of 2 N sulfuric acid and optical density (OD) values were measured at 450 nm with a reference at 630 nm [32].

### Electrophoresis and Immuno-blot analysis

CPE proteins were separated in a 10 % or 12 % SDS gel with or without 0.05 % 2-Mercaptoethanol (Sigma). To assess protein separation, the gel was stained with Coomassie brilliant blue R-250 (Sigma) according to the manufacturer's instructions. The separated proteins were transferred to a nitrocellulose membrane (Thermo Fisher, MA, USA). The membrane was then blocked with Casein/TBS and incubated with diluted anti-CPE mAbs at 4°C overnight. After washing the membrane with PBST, it was incubated with biotin-labeled anti-mouse IgE monoclonal antibodies (Chondrex) in Casein/TBS at RT for 2 hours. After washing the membrane with PBST, it was then incubated with HRP-labeled streptavidin (Chondrex) in Casein/TBS at RT for 30 minutes. The protein bands were visualized by adding TMB for immuno-blot (Thermo Fisher).

### Preparation of Monoclonal Antibody Affinity Column

A mAb affinity column was prepared following the procedures described by Sterogene Bioseparations (CA, USA). To prepare the column, mouse anti-CPE IgE mAb 2G11G7 was dissolved in 3 ml PBS at a concentration of 2 mg/ml and incubated with 1 ml ALD-Actigel and ALD-coupling solution overnight at 4°C. Unbound sites on the antibody-bound gel were blocked by incubating the gel with 0.1M Tris and ALD-coupling buffer, followed by washing with 0.1M TBS at pH 8.0. CPE in 0.1M TBS at pH 8.0 was then passed through the column and any unbound proteins were washed out with the TBS buffer. The bound fraction was eluted with 0.1M glycine-HCl buffer at pH 2.6 and then dialyzed against 0.1M TBS at pH 8.0.

### Rat basophilic leukemia cell assay

RBL-2H3 (CRL-2256) cells were purchased from the American Type Culture Collection (ATCC, VA, USA). The cells were cultured in DMEM with 15 % FCS and scraped to prepare a single-cell suspension. The cells were then resuspended at 2×10^5^ cells/ml in 0.2 ml fresh medium and plated into 96-well flat bottom tissue culture plates (Greiner). The cells were allowed to adhere for at least 3 hours. Afterwards, they were incubated with 0.2 ml fresh medium containing various concentrations of the anti-CPE IgE mAbs for 16 hours. The cells were then washed twice with PBS and challenged with 0.2 ml of various concentrations of CPE in Tyrode's buffer (137 mM NaCl, 2.7 mM KCl, 12 mM NaHCO_3_, 0.3 mM NaHPO_4_, 3 mM glucose, 0.1 % gelatin, pH 7.2). After incubating at 37°C for 1 hour, 0.1 ml of the medium was transferred to a 96-well plate (Greiner). 40 µl 1.3 mg/ml p-nitrophenyl N-acetyl-D-glucos-aminide in citrate buffer, pH 4.5 were added, and the plate was incubated at 37°C for 1 hour. The reaction was stopped by adding 45 µl of 0.5M NaOH to each well, and OD values of the formed p-nitrophenolate were measured at 410 nm. The absorption was converted into the percentage of total cellular beta-hexosaminidase content by comparison with the absorption produced by the cells which received 1 % Triton X-100 (Sigma) in Tyrode's buffer [33,34].

### A Mouse Footpad Type I Hypersensitivity Model

As a preliminary study to test the biological activities of these IgE mAbs, groups of three BALB/c mice were housed together in a cage and each mouse received 1 mg of mAbs by intravascular (IV) injection. After 24 hours, the mice were challenged with 0.1 mg of CPE or PBS by intradermal (ID) injection at their footpads. The thickness of the footpad (in millimeters) was measured using a Loop Handle Dial Thickness Gauge (Mitsutoyo, Japan).

## Results

**Fig. 1:**
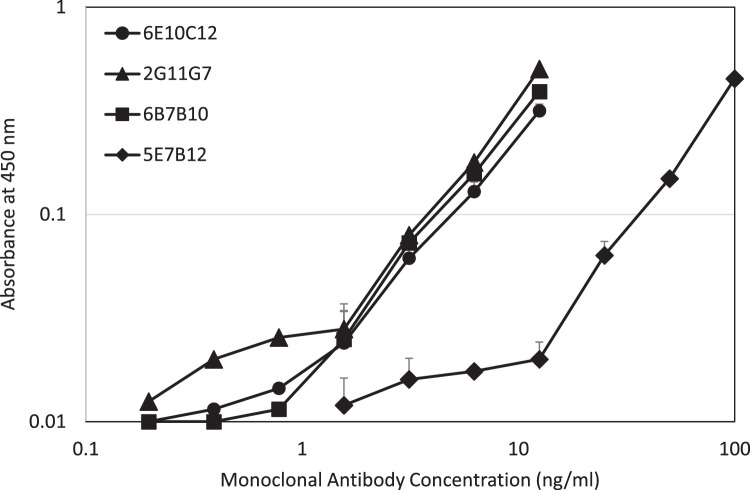
Dose dependency of each IgE monoclonal antibody against CPE in a sandwich ELISA.

After somatic cell hybridization, four hybridomas, clones 6E10C12, 2G11G7, 6B7B10, and 5E7B12 were determined to produce IgE mAbs against CPE, by screening using sandwich ELISAs employing anti-mouse IgE antibody-coated plates and biotinylated CPE as a tracer. Each IgE mAb was purified by ion-exchange chromatography from culture medium collected in a bioreactor system. In the sandwich ELISA, the four IgE mAbs reacted with biotinylated CPE dose-dependently (Fig. 1). 6E10C12, 2G11G7, and 6B7B10 showed similar dose-dependent reactivities between 1- 10 ng/ml while 5E7B12 had lower reactivities against CPE in the 10 -100 ng/ml range.

Mouse anti-CPE IgE mAbs: 6E10C12 (Circle), 2G11G7 (Triangle), 6B7B10 (Square), and 5E7B12 (Diamond), diluted with 0.1M Tris-buffered saline pH 7.5 containing 1 % BSA were added to wells coated with 10 µg anti-mouse IgE mAb, and incubated at RT for 2 hours. After washing the plates with PBST, biotinylated CPE diluted with Casein/TBS was added and incubated at RT for 1 hour. After washing the plates with PBST, avidin-peroxidase diluted with the casein buffer was added and incubated at RT for 30 minutes. After washing plates, the antibody binding was visualized with TMB at RT for 25 minutes and OD values were measured at 450 nm with a reference at 630 nm. Data is expressed as the mean ± standard deviation.

**Fig. 2:**
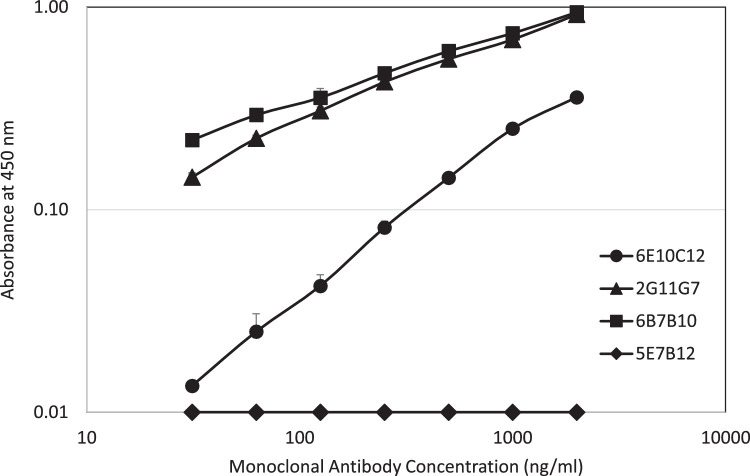
Dose dependency of each IgE monoclonal antibody against CPE in an indirect ELISA.

Then, the four IgE mAbs were evaluated by indirect ELISAs that consisted of CPE coated plates and anti-mouse IgE secondary antibodies. In the indirect ELISA, 2G11G7 and 6B7B10 demonstrated higher dose-dependent reactivities against CPE coated on the plate at between 31.5 – 2000 ng/ml. However, 6E10C12 showed lower reactivities and 5E7B12 failed to react with CPE coated on the plates in the same antibody concentration range (Fig. 2).

Anti-CPE IgE mAbs: 6E10C12 (Circle), 2G11G7 (Triangle), 6B7B10 (Square), and 5E7B12 (Diamond) diluted with 0.1M Tris-buffered saline pH 7.5 containing 1 % BSA were added to wells coated with 1 µg CPE and incubated at RT for 2 hours. After washing the plates with PBST, biotinylated anti-mouse IgE antibodies diluted with Casein/TBS was added and incubated at RT for 1 hour. After washing the plates with PBST, avidin-peroxidase diluted with Casein buffer was added and incubated at RT for 30 minutes. After washing plates, the antibody binding was visualized with TMB at RT for 25 minutes and OD values were measured at 450 nm with a reference at 630 nm. Data is expressed as the mean ± standard deviation.

**Fig. 3:**
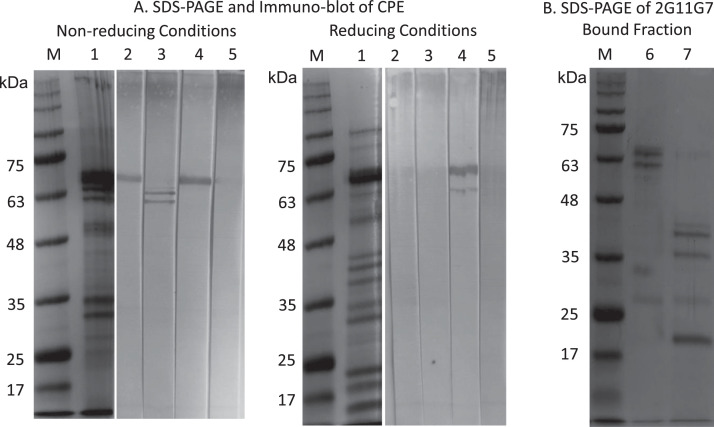
Immuno-blot analysis of anti-CPE IgE monoclonal antibodies against CPE. A. SDS-PAGE and Immuno-blot analysis of CPE under non-reducing conditions (Left), reducing conditions (Right). Coomassie brilliant blue R-250 staining; M: Molecular Marker and 1: CPE.

The protein profile of CPE was analyzed using SDS-PAGE under both reducing and non-reducing conditions. As expected, the separation of CPE antigens resulted in the detection of multiple protein bands that corresponded to Ara h 1, Ara h 2, and Ara h 3 proteins. These proteins were expected to have molecular weights of approximately 66-69 kDa, 17-19 kDa, and 57-62 kDa, respectively, when analyzed under non-reducing conditions [7]. The specific antigens of the four IgE mAbs were determined by the immune-blot analysis. Under non-reducing conditions, 6E10C12, 6B7B10, and 5E7B12 recognized a 68 kDa protein, while 2G11G7 bound to both 63 and 60 kDa proteins. However, under reducing conditions only 6B7B10 recognized both 68 and 63 kDa proteins (Fig. 3A). In fact, the 68 kDa band was recognized by 3A1E11 IgM mAb created by immunization with the GERTRGRQPGDYDD (Ara h 1: 89-102) peptide in immuno-blot analysis under non-reducing conditions (Data not shown).

Of the four mAbs, 2G11G7 was selected for further investigation to verify its antigen. An immuno-affinity column was prepared using 2G11G7, and CPE was passed through the column to obtain the bound fraction. The fraction was analyzed by SDS-PAGE under reducing and non-reducing conditions. Under non-reducing conditions, the fraction showed bands at 65 and 60 kDa, which disappeared under reducing conditions. In contrast, major bands at 42, 37, and 20 kDa were observed under reducing conditions (Fig. 3B). N-terminal amino acid analysis of the 3 bands is highly matched with Ara h 3 (1-5 RQQPE: 42 and 37kDa, and 323-328 GIEET: 20kDa).

Immuno-blot; 2: 6E10C12, 3: 2G11G7, 4: 6B7B10, and 5: 5E7B12

B. SDS-PAGE analysis of 2G11G7 affinity purified samples under 6: non-reducing conditions and 7: reducing conditions.

**Fig. 4:**
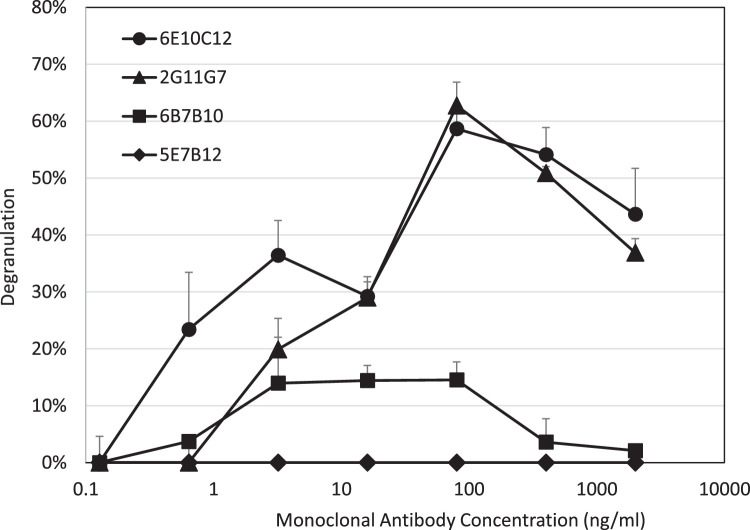
RBL-2H3 cell degranulation with CPE and anti-CPE IgE monoclonal antibodies.

The biological activities of the four IgE mAbs were evaluated by beta-hexosaminidase release from RBL-2H3 cells in degranulation assays. The RBL-2H3 cells were sensitized with serial dilutions (0.128 - 2000 ng/ml) of the IgE mAbs and were then challenged with 2 µg/ml CPE. A typical prozone effect was observed with a bell-shaped dose–response curve. With 2G11G7 and 6E10C12, the degranulation peaked at 80 ng/ml of IgE mAbs, ranging from approximately 20 % to 60 %. At concentrations of 0.128 ng/ml IgE mAbs, beta-hexosaminidase release approached background levels. With 6B7B10, the degranulation peaked at between 3.2 and 80 ng/ml of IgE mAb, ranging from approximately 3 to 15 %. Interestingly 5E7B12 failed to degranulate RBL-2H3 cells (Fig. 4).

RBL-2H3 cells were cultured in 0.1 ml DMEM containing 15 % FBS at 10^6^ cells/well in a 96-well plate for 3 hours, and then treated with anti-CPE IgE mAbs: 6E10C12 (Circle), 2G11G7 (Triangle), 6B7B10 (Square), and 5E7B12 (Diamond) at 37°C for 16 hours. After washing cells with PBS two times, 2 µg/ml CPE in Tyrode's buffer was added at 0.2 ml/well and then were incubated at 37°C for 1 hour. 0.1 ml medium from each well was transferred to a 96-well plate and assayed for beta-hexosaminidase activity. The degranulation of RBL-2H3 cells was expressed as a ratio compared with 100 % degranulation of the cells which received 1 % Triton-X in Tyrode's buffer. Data is expressed as the mean ± standard deviation.

**Fig. 5:**
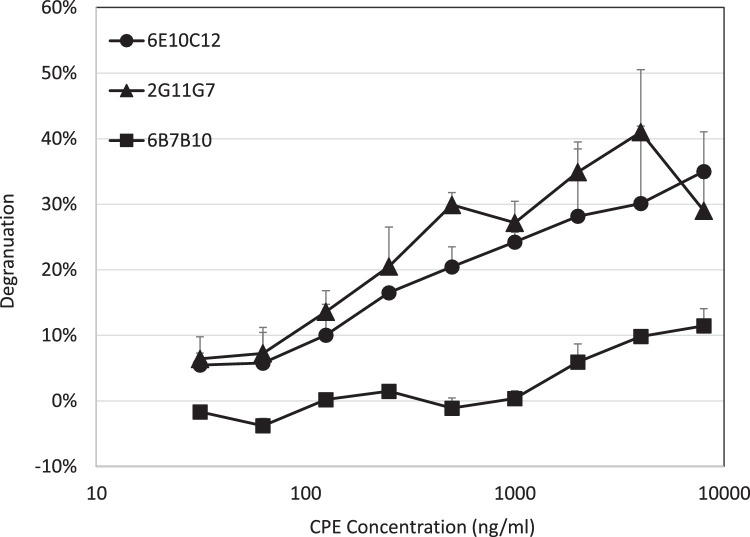
Dose dependency of CPE in RBL-2H3 cells degranulation with Anti-CPE monoclonal antibodies. RBL-2H3 cells were cultured in 0.1 ml of DMEM containing 15 % FBS at 10^6 cells/well in a 96-well plate for 3 hours, and then treated with anti-CPE IgE mAbs: 6E10C12 (Circle), 2G11G7 (Triangle), 6B7B10 (Square), and 5E7B12 (Diamond) at 37°C for 16 hours. After washing cells with PBS two times, various concentrations of CPE in Tyrode's buffer were added at 200 µl/well and then incubated at 37°C for 1 hour. 0.1 ml of medium from each well was transferred to a 96-well plate and assayed for beta-hexosaminidase activity. The degranulation of RBL-2H3 cells was expressed as a ratio compared with 100 % degranulation of the cells which received 1 % Triton-X in Tyrode's buffer. Data is expressed as the mean ± standard deviation.

The dose dependency of CPE concentration for sensitization of RBL-2H3 cells was evaluated with the three responding anti-CPE IgE mAbs. Sensitizing with CPE at 100 ng/ml, both 2G11G7 and 6E10C12, induced degranulation dose-dependently from 500 to 8000 ng/ml. For 6B7B10, CPE sensitization at 20 ng/ml 6B7B10 showed degranulation between 200 and 8000 ng/ml (Fig. 5).

**Fig. 6:**
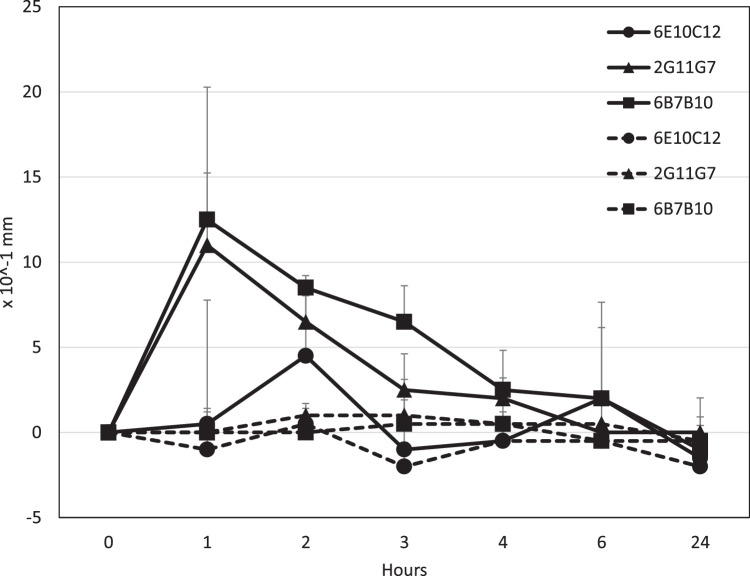
Footpad type I hypersensitivity by anti-CPE IgE monoclonal antibodies.

Three anti-CPE IgE mAbs were evaluated for mouse footpad type I hypersensitivity. Each mouse was given an IV injection with 1 mg of each IgE mAb, followed by a CPE injection in the footpad. Mice given 2G11G7 and 6B7B10 IgE mAbs developed paw swelling, 1.10 +/- 0.42 mm and 1.25 +/- 0.78 mm respectively, that peaked at 1-2 hours after the CPE injection and resolved to base levels within 6 hours. However, 6E10B10 failed to induce paw swelling. In contrast, mice that received anti-CPE IgE mAbs and PBS did not exhibit any paw swelling (Fig. 6).

The footpad thickness of mice after IV administration of 1 mg of mAbs: 6E10C12 (Circle), 2G11G7 (Triangle), 6B7B10 (Square), followed by ID administration of 0.1 mg CPE (Solid line) or PBS (Dashed line) at the footpad. Data is expressed as the mean ± standard deviation.

**Fig. 7:**
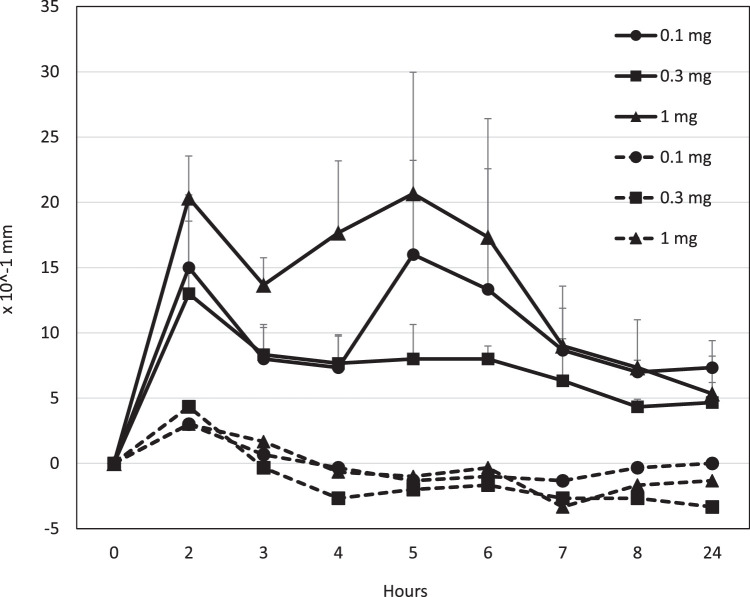
Dose dependency of footpad type I hypersensitivity by monoclonal antibody, clone 2G11G7. The footpad thickness of mice after IV administration of each dose of mAbs: 0.1 mg (Circle), 0.3 mg (Square), and 1 mg (Triangle), followed by a 0.1 mg CPE (solid line) or PBS (dashed line) ID administration at the footpad. Data is expressed as the mean ± standard deviation.

The dose-dependency of footpad type I hypersensitivity induced by mAb 2G11G7 was evaluated. Mice given CPE and 0.1, 0.3, or 1 mg of mAb 2G11G7 developed paw swelling, 1.30 +/- 0.43 mm, 1.60 +/- 0.13 mm, and 2.07 +/- 0.10 mm, respectively. The paw swelling lasted for 6 hours at all doses and resolved to base levels within 8 hours after CPE injection at the footpad. In contrast, mice that received IgE mAbs and PBS did not exhibit any paw swelling (Fig. 7).

## Discussion

In the present study, we established four mouse hybridoma clones that produce IgE mAbs against peanut antigens. The IgE mAbs showed different reactivity patterns in two ELISA systems: the sandwich ELISA and indirect ELISA (Fig. 1 and 2). In the indirect ELISA, 2G11G7 and 6B7B10 showed high reactivity, while 6E10C12 showed only 10 % reactivity and 5E7B12 failed to react to the coated CPE antigens. On the other hand, 2G11G7, 6B7B10, and 6E10C12 showed similar reactivity in the sandwich ELISA. These reactivity differences can be explained by the epitopes on the antigens. When plates are coated with antigens, the hydrophobic sites in the antigens tend to attach to the plate surface. This coating condition can interfere with antibody binding because epitope sites may not be exposed. In contrast, the sandwich ELISA uses biotinylated CPE as a tracer which should not interfere with epitope exposure. As a result, the sandwich ELISA can provide a more accurate reflection of antibody reactivity against antigens.

The IgE mAbs recognized their antigens in immuno-blot analysis under non-reducing conditions, indicating that they recognize linear epitopes that remain stable even under heat denaturation at 100°C (Fig. 3A). Based on the resulting in antigen's molecular weight, 6B7B10, 6E10C12, and 5E7B12 likely recognize the Ara h 1 protein, which has a molecular weight of 63 kDa. Under reducing conditions, 6B7B10 and 5E7B12 failed to recognize the Ara h 1 protein. Although Ara h 1 does not contain internal disulfide bonds, the epitopes recognized by 6B7B10 and 5E7B12 may be affected by reducing conditions. However, 6E10C12 bound to 68 kDa and 63 kDa bands. The 63 kDa band must be analyzed to understand the epitope of 6E10C12. Discrepancies in estimated molecular weights based on amino acid composition and molecular weight in SDS-PAGE sometimes occur depending on the molecular weight markers and SDS-PAGE conditions.

Ara h 1 and Ara h 3 exist in homotrimer (and hexamer) in physiological conditions that were used for oral immunization. Their monomeric or trimeric form and native or denatured conditions may affect mAb reactivities by exposing their epitopes in the antigen. Since there are discrepancy in indirect ELISA using CPE and purified allergens and immuno-blot, further investigations will be needed to understand these discrepancies among assays. 2G11G7 likely binds to the Ara h 3 protein as shown in immune-blot analysis under non-reducing conditions (Fig. 3A). We specifically identified the antigen as Ara h3 using affinity chromatography analysis with 2G11G7, as confirmed by SDS-PAGE (Fig. 3B) and N-terminal amino acid analysis of the bands. Ara h 3 has been identified as a major or minor allergen, depending on the population sampled as it triggers an IgE-mediated reaction in 44−77 % of the peanut allergy patient population [35], [36], [37]. Ara h3 is a seed storage protein belonging to the 11S globulin family. It exists as a trimer (or hexamer) consisting of identical 58 kDa subunits, with molecular masses of 180 or 360 kDa, respectively [38,39]. Each subunit is derived from a single precursor which is post-translationally cleaved to produce an acidic and a basic chain, ranging from 14 to 45 kDa, that are held together by a disulfide bond [40], [41], [42]. Ara h3 shows two isotypes of 60 kDa and 54 kDa bands in non-reducing conditions due to proteolytic cleavage of the C-terminal region of the acid subunits [42]. However, under reducing conditions, 2G11G7 failed to recognize Ara h3 proteins indicating that the epitope of the mAb must involve the complex site of basic and acidic subunits, including the disulfide bonds (85 and 329)[39]. Interestingly, Ara h4 has 91.3 % sequence homology with Ara h3 [43] and is considered to be the same allergen[42,44]. This raises the possibility that Ara h4 may also work as an allergen to initiate an allergic reaction, including MC activation by 2G11G7.

The biological activities of the IgE mAbs were evaluated by measuring the degranulation of RBL-2H3 cells. The results showed a bell-shaped dose–response curve, with three mAbs capable of degranulating the cells. Specifically, 2G11G7 and 6E10C12 had peak degranulation levels of 60 % at 80 ng/ml, while 6B7B10 peaked at 15 % at 12.5 ng/ml. This bell-shaped reaction has been reported previously with an anti-Dinitrophenyl (DNP) IgE antibody [45]. An antigen recognized by IgE antibodies must have at least two identical epitopes in a molecule, to allow them to bridge two IgE antibodies bound on IgE receptors on the MCs. The peaked shape of RBL-2H3 cell activation was observed based on the IgE concentration. At high concentrations of multivalent antigens, monovalent complexes form with each IgE antibody on its IgE receptor, preventing the bridging of two IgE antibodies. As a result, high concentrations of antigens would elicit lower degranulation due to crowding of the antibodies by antigens. 5E7B12 failed to trigger degranulation in RBL-2H3 cells, even though in the sandwich ELISAs, it was able to bind to free-floating CPE. This can be due to 5E7B12 being selectively recognized by mouse FcεRI receptors, but not rat receptors or possibly by low-affinity FcεRII receptors, but not high-affinity FcεRI receptors [23]. It is important to note that RBL-2H3 is a basophilic leukemia cell line isolated and cloned from Wistar rat basophilic cells. These cells only express high-affinity FcεRI receptors on their cell surface [46].

In degranulation assays, a high concentration of CPE demonstrated partial RBL-2H3 cell degranulation (data not shown). As a result, a CPE concentration of 2 µg/ml was chosen, as it showed low self-activation and effectively degranulated RBL-2H3 cells in the presence of IgE. The degranulation of RBL-2H3 cells by CPE alone may be a result of the inherent presence of Lipopolysaccharides (LPS) in CPE [47]. RBL-2H3 cells express innate receptors, Toll-like receptor (TLR) 2 and TLR4, on their cell surface. Despite washing cells with PBS, residual serum components such as adaptor protein myeloid differentiation 88 (MyD88) and CD14 in the wells can form a TLR4 complex [48]. This complex can be activated by LPS in CPE, triggering intracellular signaling and degranulating the cells [14,15].

In type I hypersensitivity reaction studies, the development of footpad swelling was observed in mice who received 2G11G7 and 6B7B10, but not in those who received 6E10C12. These findings revealed an inconsistency with the results from the RBL-2H3 cell degranulation assays in which 6E10C12 was able to degranulate RBL-2H3 cells but failed to induce type I hypersensitivity in mice. It is possible that 6E10C12 has a higher affinity for rat IgE receptors, but lower affinity for mouse IgE receptors, despite all four hybridomas being generated using mouse spleen cells and Sp2/0-Ag14 myeloma cells. Thus, it may be crucial to use mouse MCs with 6E10C12 to determine its ability to degranulate mouse MCs and induce delayed type I hypersensitivity *in-vivo* [16]. Further investigation is necessary to understand this discrepancy between the cell-based assays and mouse studies for the 6E10C12 mAb.

It Is important to note that while 6B7B10 had a lower activation rate of only 15 % in cell degranulation assays, its peak activation concentration was lower than that of the other mAbs. Interestingly, 6B7B10 was able to induce a similar level of footpad type I hypersensitivity as 2G11G7. These results indicate that it is necessary to consider the total activation capacity by determining both the optimal concentration and the activation rate when evaluating the biological activities of these mAbs in *in-vivo* studies. In conclusion, 2G11G7 is the most suitable IgE antibody for studying CPE-related allergic reactions in *in-vitro* and *in-vivo* assays. 6E10C12 can be used as a negative control in *in-vivo* studies, while 5E7B12 works as a negative control in mast cell degranulation assays and indirect ELISAs.

Despite all the IgE mAbs showing positive results in immuno-blot analysis, three out of the four mAbs failed to bind to heat denatured CPE in indirect and sandwich ELISAs, as well as failing RBL-2H3 cell degranulation assays with heat-denatured CPE (data not shown). Additionally, it is worth noting that upon heating, Ara h 1 and Ara h 3 may form aggregates, which can decrease their solubility and alter their structure [49]. As a result, epitopes of mAbs may be masked. In preparing foods containing peanuts, boiling or heating is often involved. Therefore, further investigation is needed to evaluate the biological functions of our IgE mAbs using CPE from roasted peanuts or processed foods as we had used CPE from unroasted (native) peanuts in our analysis. Some researchers use lightly roasted peanuts in the preparation of their CPE. It is important to consider the preparation protocols used for the CPE in evaluating the reactivity and biological activity of our IgE mAbs [50], [51], [52], [53], [54].

A summary of the applications of the IgE mAbs are shown in Table 1. Out of four anti-CPE IgE mAbs, two IgE mAbs can be used in both *in-vitro* and *in-vivo* peanut allergy studies. These mAbs can induce acute allergic reactions with only sensitization in mice, making them useful for evaluating therapeutic methods related to mast cell activation. The use of these mAbs eliminates the need for lengthy immunization protocols, streamlining the process of allergic studies. These novel anti-CPE IgE mAbs, along with the protocols outlined in this article, offer valuable guidance for studying allergic reactions across various platforms, thereby advancing peanut allergy research.

**Table 1:** Summary of the Application of Anti-CPE IgE Monoclonal Antibodies.

Clone name/Application	6E10C12	2G11G7	6B7B10	5E7B12
Immuno-blot analysis	Moderate	High	High	Moderate
Indirect ELISA	Moderate	High	High	None
Sandwich ELISA	High	High	High	Moderate
RBL-2H3 cell degranulation	High	High	Moderate	None
Footpad type I hypersensitivity reaction	Low - None	High	High	ND

However, we must pay attention to some considerations. In *in-vitro* studies, although ELISA is effective for validating IgE mAbs to Ara allergens, assay results must be considered with their epitope, sensitivities and specificities. Furthermore, when assessing biological activities of mouse IgE mAbs to mouse or rat MCs, variations may arise by affinities between IgE mAbs and IgE receptors affecting to cell-based assays (or *ex-vivo* assays) and *in-vivo* assays. In *in-vivo* studies, this protocol has limited efficacy in studying the interaction between IgE and MCs during the allergic acute phase, as IgE may only remain in tissues in a short period. For the investigation of T-cell responses and chronic phases of allergic reactions, conventional oral immunization methods using CPE or allergens must be used. Additionally, there are discrepancies between mouse and human allergic reactions depending on mouse strains and environmental factors [11,^55^,56]. To minimize these differences, humanized mice can be used to reduce discrepancies between species for better understanding allergic reactions [57].

Nonetheless, the use of mAbs ensures high reproducibility compared to serum or polyclonal antibodies, as they do not exhibit batch-to-batch variations. Although effective utilization of IgE mAbs should take these considerations and limitations into account the IgE mAbs can still serve as valuable tools, especially in specific studies like inhibitor screening in MC activation.

## CRediT authorship contribution statement

**Takaki Waritani:** Conceptualization, Methodology, Investigation, Data curation, Writing – original draft. **Sidney Lomax:** Investigation. **Dawn Cutler:** Investigation. **Jessica Chang:** Validation, Writing – review & editing.

## Declaration of Competing Interests

The authors declare that they have no competing interests.

## Data Availability

Data will be made available on request.
